# The haemodynamic assessment of patients with pulmonary arterial hypertension

**DOI:** 10.21542/gcsp.2020.4

**Published:** 2020-04-30

**Authors:** Stefano Ghio

**Affiliations:** Division of Cardiology, Foundation “I.R.C.C.S. Policlinico San Matteo”, Pavia, Italy

## Introduction

Right heart catheterization (RHC) has a crucial role in the management of patients with pulmonary hypertension (PH). Firstly, it is mandatory in subjects with the suspicion of PH to confirm the diagnosis. Secondly, it is strongly recommended in the follow-up of patients with confirmed pulmonary arterial hypertension (PAH) to evaluate drug efficacy and in case of clinical deterioration^1^.

In order to always perform a complete and correct RHC, clinicians carrying out the examination must be guided not only by technical recommendations, but also by the accurate knowledge of the different clinical issues which have to be addressed in these different situations (Table 1).

## Best practice guidance

RHC can be a challenging procedure in patients with PH and requires expertise, attention to detail, and meticulous collection of data^2^.

Whenever possible, RHC should be performed in stable, non-acute clinical conditions. Although any systemic large vein may be used for venous access^3,4^, the femoral and internal jugular veins are most commonly used in clinical practice. The femoral access is easily compressible and complications (e.g., pseudoaneurysm, arteriovenous fistula, or retroperitoneal bleeding) are more likely when arterial and venous access are both obtained. The main disadvantage is the need for bed rest after the procedure.

The internal jugular vein allows easy access to the pulmonary artery. Complications are uncommon using an ultrasound-guided approach which gives the possibility to minimize the risks of the procedure, visualizing the location of the jugular vein (lateral or anterior or medial to the carotid artery) and the direction of the needle.

A wrong zero-level set is among the most common mistakes and a major confounding factor during RHC. All pressure measurements are the difference between the pressure at the chosen zero level and the pressure within the cardiac chamber or vessel where the catheter tip is positioned^5^. A zero-level set five cm above (or below) the midthoracic level results in underestimation (or overestimation) of all pressures by approximately four mmHg. This may significantly affect management of patients.

The zero reference defines the location in the circulatory system where changes in body position do not affect pressure measurements. The current consensus is that pressure transducer should be zeroed at the mid-thoracic level (midpoint between anterior sternum and bed surface) with the patient supine, which best approximates the level of the right atrium. These references should be maintained for the entire duration of the procedure.

The thermodilution technique can be considered the preferred method of cardiac output monitoring, even among patients with very low cardiac output and/or severe tricuspid regurgitation. Thermodilution has in fact proven to be a reliable method when compared with the direct Fick measurement; on the contrary, the indirect Fick method is less reliable than the other techniques.

**Table 1:** Clinical objectives of RHC in PH/PAH.

**Main clinical issues to be addressed by RHC**	In the diagnostic phase	During follow-up
Is PH present?	**Hemodynamic definition = mean PAP >20 mmHg and PVR ≥3 WU.**	
Is PH pre or post-capillary?	**Accurate measure of PAWP is crucial (sometimes LVEDP is required).Fluid challenge may be useful.Agreement with pre-test clinical evaluation and imaging data is necessary.**	
Is PH causing right ventricular failure?	**RAP >14 mmHg, CI <2.0 l/min/m^2^ and SvO2 <60% defines a situation of RV failure and thus characterize the highest risk patients**	
Which is the risk of the patient?		**Low-risk cutoffs: RAP <8 mmHg;CI ≥2.5 l/min/m^2^; SvO2 >65%Intermediate-risk cutoffs: RAP 8–14 mmHg; CI 2.0–2.4 l/min/m^2^; SvO2 60–65%High-risk cutoffs: RAP >14 mmHg; CI <2.0 l/min/m^2^; SvO2 <60%**
Is pharmacological treatment effective?		**Based on assessment of changes not only in PVR but also in CI (or SVi) and RAP.**

In order to cope with the intrinsic random errors of pressure and flow determinations made with a fluid-filled pulmonary artery catheter, it is now clearly recommended that measurements should be repeated in triplicate to obtain values within a 10% agreement and averaged^6,7^.

### Which are the clinical issues to be addressed by the diagnostic RHC?

• Is PH present?
• Is PH pre-capillary or post-capillary?
• Does PH cause right ventricular failure?

#### Is PH present?

The hemodynamic definition of PH is based on the value of mean pulmonary arterial pressure (mPAP) which must be ≥25 mmHg at rest according to 2015 ESC/ERS Guidelines. This can only be obtained at RHC. Doppler echocardiography is used to estimate the right ventricular (RV) systolic pressure by estimating the pressure gradient between the right ventricle and the right atrium using the modified Bernoulli equation; an estimate of right atrial pressure obtained by the evaluation of dimensions and collapsibility of the inferior vena cava must be added to this number to approximate the RV systolic pressure, which coincides with the systolic pulmonary artery pressure (sPAP) in the absence of pulmonic stenosis.

Numerous methods have been suggested to derive non-invasively mPAP by sPAP^8,9^, however, these have not gained clinical acceptance in international guidelines. In general, Doppler echocardiography allows for accurate measurements of the pulmonary circulation, but has only moderate precision, which explains why echocardiographic estimates of pulmonary pressures are valid for population studies, but cannot be used for the individual diagnosis of pulmonary hypertension^10^.

In addition, the relationship between the systolic and the mean pressure in a vessel is not linear but it is highly dependent on the stiffness of the vessels, which may vary from patient to patient and cannot be determined non-invasively.

A new hemodynamic threshold for PH has been proposed by the 6th WSPH Task Force on PH diagnosis and classification (combination of mPAP >20 mmHg and pulmonary vascular resistance (PVR) ⩾3 Wood Units)^11^. Including pulmonary vascular resistance in the definition of pre-capillary PH is essential, allowing discrimination of elevation of PAP due to pulmonary vascular disease from those due to elevation of PAWP due to high cardiac output. This new definition reinforces the necessity of using the RHC for a precise definition of presence/absence of PH.

#### Is PH pre-capillary or post-capillary?

Accurate measurement of left atrial pressure is essential for distinguishing pre-capillary from post-capillary PH, i.e., patients with left heart disease. In particular, heart failure with preserved ejection fraction (HFpEF) is most frequently misclassified as PAH and thus erroneously treated with PAH-specific therapy. Pulmonary artery wedge pressure (PAWP) is commonly used as a surrogate of left atrial pressure; in the absence of mitral stenosis, PAWP measured at end-diastole (i.e., typically as the mean of the a-wave or, alternatively, a QRS-gated approach) more closely approximates left ventricular end-diastolic pressure (LVEDP)^12,13^. LVEDP direct measurement is only used when an accurate PAWP cannot be obtained^14^.

PAWP is obtained using a Swan-Ganz catheter with the balloon inflated in a branch pulmonary artery, preventing blood flow or the transmission of pressure from the proximal pulmonary arteries. The static column of blood transmits left atrial pressure to the catheter tip, allowing an estimate of left atrial pressure. Obtaining a reliable PAWP requires expertise and attention. It is important to achieve a stable balloon occlusion position, avoiding under-wedging or over-wedging.

Pressure measurements are to be carefully recorded at end expiration, ensuring that the patient does not produce a Valsalva manoeuvre. All pressure values, including PAWP, are recorded as the mean of 3 to 5 measurements obtained at the end of normal expiration. This guideline recommendation may not be applicable in patients with chronic obstructive pulmonary disease (COPD), in whom there is often a prominent swing in intrathoracic pressure affecting intracardiac pressures. Averaging PAWP over several respiratory cycles may be the most reasonable compromise to compensate for respiratory fluctuations as positive expiratory and negative inspiratory intrathoracic pressures cancel each other out^6^.

There are several tricks to be sure that an accurate PAWP is obtained, including: PAWP value should be equal to or lower than diastolic pulmonary arterial pressure, the PAWP waveform must exhibit clear A and V waves, a respiratory swing should be visible, blood sampling from the distal lumen of the catheter should detect an oxygen saturation in the occlusion position similar or higher than the systemic saturations.

However, expert clinicians must be aware of the fact that the optimal PAWP threshold for distinguishing pre-capillary from post-capillary PH remains a matter of debate. Although the 2015 ESC/ERS guidelines recommend a threshold of 15 mmHg, values between 10 and 18 mmHg have also been suggested^15–19^. The first randomized, multicenter trial comparing the effects of the continuous intravenous infusion of epoprostenol plus conventional therapy vs, conventional therapy alone in patients with severe primary pulmonary hypertension included patients with PAWP ≤12 mmHg^16^.

In the REVEAL registry, a cutoff of 18 mmHg was used to avoid misclassifying older patients with PAH or patients with pulmonary vascular disease and a minor degree of left ventricular diastolic dysfunction^19^. In general, high cutoff risks misclassifying HFpEF patients as PAH, whereas a lower threshold carries the opposite risk. Therefore, most importantly, the hemodynamic values recorded at the diagnostic RHC must be in accordance with the clinical picture and with the non-invasive information^20^. This means that a complete and accurate diagnostic RHC is always preceded by a clinical evaluation of the patient and by a focused echocardiographic and Doppler examination to have a pre-test probability of PH due to left heart disease. The clinician performing the diagnostic RHC must correctly interpret the hemodynamic values recorded during the catheterization.

Importantly, PAWP is not a constant value, but a dynamic parameter affected by the afterload of the left ventricle and by fluid balance. In many patients with left heart disease, PAWP can be lowered to less than 15 mmHg with intense diuretic treatment and vasodilator administration. Therefore, if the *a priori* probability of post-capillary PH is high (due to the presence of risk factors or to the presence of an enlarged left atrium and of diastolic dysfunction at echocardiography) and a low PAWP is recorded, RHC must include a fluid challenge test to uncover latent HFpEF^21^.

In the opposite situation, i.e., a high pre-test probability of pre-capillary PH and recorded PAWP values above 15 mmHg, the clinician should be prompted to re-check the zero level and to wedge the catheter in different positions.

In case of post-capillary PH, this should be classified as: 1) isolated PH (IpcPH): PAWP >15 mmHg and mPAP >20 mmHg and PVR <3 WU; or 2) combined PH (CpcPH): PAWP >15 mmHg and mPAP >20 mmHg and PVR ⩾3 WU^12^.

Finally, in patients with idiopathic, heritable or drug-related PAH, the diagnostic RHC must include an acute pulmonary vasoreactivity test in order to identify patients suitable for calcium channel blocker therapy. Vasoreactivity testing is usually performed with inhaled nitric oxide (10 to 20 parts per million). A positive test is defined as a reduction of mPAP ≥10 mmHg from baseline to an absolute value of mPAP ≤40 mmHg with unchanged or increased cardiac output^1^.

#### Is PH causing right ventricular failure?

In the 1980s, the National Institutes of Health (NIH) Registry on Primary Pulmonary Hypertension demonstrated that mortality in incident PAH patients best correlates with two hemodynamic variables obtained at the diagnostic RHC which describe RV function: i.e., one indicator of pump function (cardiac index, CI) and one indicator of systemic congestion (right atrial pressure, RAP)^16^.

The latest recommended treatment algorithm for incident PAH patients foresees choice between two options. Patients at low or intermediate risk should be treated with initial oral combination therapy with an ERA and a PDE5i (monotherapy is limited to specific PAH subsets).

In high-risk patients, combination therapy including i.v. prostacyclin analogues is recommended.

Although risk assessment in PAH is complex and must be based on a multidimensional evaluation of the patients, what is necessary in incident patients is only to identify the highest risk subgroup. This is easy using the hemodynamic parameters obtained at RHC: RAP >14 mmHg, CI <2.0 l/min/m^2^ and SvO_2_ <60% defines a situation of RV failure and thus characterize the highest risk patients.

### Which clinical issues are to be addressed by RHC during the follow-up of PAH patients?

• What is the risk of the patient?
• Is pharmacological treatment effective?

#### Risk stratification

The assessment of the prognosis has always been considered an important part of the evaluation of PAH patients since the publication of the NIH registry nearly three decades ago, demonstrating the strong association with mortality of a low CI and of an elevated RAP^16^. Over time, different tools have been proposed to predict outcome, but these two hemodynamic variables maintained a pivotal role in the assessment of prognosis in PAH^22–26^.

The 2015 ESC/ERS PH guidelines introduced the recommendation of regular and comprehensive risk assessment at expert centers using a range of invasive and non-invasive parameters; the guidelines in fact stated that the goal of treatment in patients with pulmonary arterial hypertension (PAH) is to achieve or maintain a low-risk clinical profile.

The complexity of the 2015 ESC/ERS chart for risk assessment was overcome in 2017, when novel abbreviated versions of the ESC/ERS risk stratification model were evaluated retrospectively in newly diagnosed cohorts of PAH patients demonstrating that it is possible to stratify patients into low, intermediate and high risk of events^27–29^.

As expected, hemodynamic parameters play a relevant role in these tools. Importantly, haemodynamic parameters at the time of follow-up RHC are also independently associated with better long-term outcome. In particular, there is additional value in quantifying the stroke volume index (SVi) from a follow-up RHC; patients with a SVi <38 ml⋅m^2^ have worse long-term outcomes even when multiple low-risk criteria are present^30,31^.

An intense debate is ongoing in the literature on the role of non-invasive markers of prognosis and whether these could eventually replace the invasive hemodynamic assessment^32,33^. In the study from the French registry, brain natriuretic peptide (BNP) or N-terminal (NT)-pro-BNP measurements, NYHA functional class evaluation and exercise capacity at 6MWD allowed to identify a subgroup at low-risk, having a 100%, 99% and 97% survival at 2, 3 and 5 years, respectively.

The usefulness of non-invasive methods to identify low-risk groups was confirmed in the COMPERA registry, with 1-, 3-, and 5-year survival rates of 100%, 100% and 95%, respectively^34^. However, a main issue is that only a small minority of patients meets all these noninvasive criteria during follow-up (9% in the COMPERA study and 19% in the French study). Thus, most patients (who are classified in an intermediate risk condition) should perform RHC to better elucidate pulmonary haemodynamics and right ventricular function, thus clarifying their risk.

One could argue that non-invasive imaging techniques, either magnetic resonance imaging (MRI) or echocardiography, being able to provide a direct evaluation of the right heart structure and function, should be superior to any indirect evaluation of the right ventricular function provided by hemodynamic parameters. As a matter of fact, the prognosis of PAH patients is determined by the impact of the elevated afterload on the function of the right ventricle^35–37^.

There is no doubt in the literature that assessment of right ventricular function with MRI is of great prognostic utility in PAH^38–40^. The usefulness of echocardiography is more controversial, possibly because it is considered a less robust technique than MRI, despite the extensive literature reporting that a great number of echocardiographic variables may provide significant prognostic information^41–46^. Currently, the only echocardiographic parameters included in the risk assessment chart of the ESC/ERS 2015 guidelines are pericardial effusion and right atrial area. In summary, the weight of the evidence available at the current time favours the utility of RHC for routine follow-up of PAH patients.

We believe that in the future routine RHC may not be necessary during follow-up for specific subgroups of patients, possibly those who meet noninvasive low-risk criteria, but this has yet to be demonstrated.

#### Evaluation of drug efficacy

The issue of evaluation of drug efficacy is strictly linked with the issue of risk stratification. Treatment of PAH patients cannot be considered as a simple prescription of drugs, but it is a complex strategy that includes an initial multidimensional evaluation of severity of the disease and the subsequent response to treatment; thus, RHC is an important part in the evaluation of response. In fact, the goal of treatment in patients with PAH is achieving a low-risk status, which, hemodynamically, is characterized by a RAP <8 mmHg and a CI ≥2.5 l/min/m^2^.

Not unexpectedly, RHC has been used to test drug efficacy in the seminal randomized, controlled trials for currently approved PAH therapies. In these trials the hemodynamic parameters used as end points (not necessarily primary end-points) were PVR, CI and RAP. Other additional hemodynamic parameters describing RV function have been occasionally used to define drug efficacy, such as RV stroke work, RV power or pulmonary arterial compliance but none has so far been validated.

## Conclusions

In PAH patients, RHC is mandatory to establish the presence and the hemodynamic type of PH. It is also extremely important during the follow-up of PAH patients to evaluate the risk of the patients, to guide therapy and to assess efficacy of treatments.

Performing a correct and complete RHC in PAH requires not only knowledge and expertise in the procedure itself but, additionally, knowledge and expertise in the different clinical issues to be addressed in the different clinical situations in which RHC is necessary.
